# eHealth Search Patterns: A Comparison of Private and Public Health Care Markets Using Online Panel Data

**DOI:** 10.2196/jmir.6739

**Published:** 2017-04-13

**Authors:** Janina Anne Schneider, Christopher Patrick Holland

**Affiliations:** ^1^ Alliance Manchester Business School University of Manchester Manchester United Kingdom; ^2^ Information Systems Group University of Münster Münster Germany

**Keywords:** health information management, medical informatics, information science

## Abstract

**Background:**

Patient and consumer access to eHealth information is of crucial importance because of its role in patient-centered medicine and to improve knowledge about general aspects of health and medical topics.

**Objectives:**

The objectives were to analyze and compare eHealth search patterns in a private (United States) and a public (United Kingdom) health care market.

**Methods:**

A new taxonomy of eHealth websites is proposed to organize the largest eHealth websites. An online measurement framework is developed that provides a precise and detailed measurement system. Online panel data are used to accurately track and analyze detailed search behavior across 100 of the largest eHealth websites in the US and UK health care markets.

**Results:**

The health, medical, and lifestyle categories account for approximately 90% of online activity, and e-pharmacies, social media, and professional categories account for the remaining 10% of online activity. Overall search penetration of eHealth websites is significantly higher in the private (United States) than the public market (United Kingdom). Almost twice the number of eHealth users in the private market have adopted online search in the health and lifestyle categories and also spend more time per website than those in the public market. The use of medical websites for specific conditions is almost identical in both markets. The allocation of search effort across categories is similar in both the markets. For all categories, the vast majority of eHealth users only access one website within each category. Those that conduct a search of two or more websites display very narrow search patterns. All users spend relatively little time on eHealth, that is, 3-7 minutes per website.

**Conclusions:**

The proposed online measurement framework exploits online panel data to provide a powerful and objective method of analyzing and exploring eHealth behavior. The private health care system does appear to have an influence on eHealth search behavior in terms of search penetration and time spent per website in the health and lifestyle categories. Two explanations are offered: (1) the personal incentive of medical costs in the private market incentivizes users to conduct online search; and (2) health care information is more easily accessible through health care professionals in the United Kingdom compared with the United States. However, the use of medical websites is almost identical, suggesting that patients interested in a specific condition have a motivation to search and evaluate health information, irrespective of the health care market. The relatively low level of search in terms of the number of websites accessed and the average time per website raise important questions about the actual level of patient informedness in both the markets. Areas for future research are outlined.

## Introduction

### Background

The Internet hosts over 100,000 health and medical-related websites, termed as eHealth websites in this study. In the United States, 95 million adults, about 80% of all adult Internet users, have searched for eHealth-related information [1]. Retrieval of eHealth information is one of the most common reasons for accessing the Internet [1] and it is estimated that 4.5% of total Internet searches worldwide are related to eHealth [2,3]. Almost 7 million eHealth searches are conducted on *Google.com* each day [1,4,5]. It has been shown that most online health searches are guided by finding concrete answers to specific questions, for example, to obtain information about treatments, symptoms, diseases, and conditions [6]. In a detailed study of online users of NHS Direct, using survey and detailed interviews, four main motivations to search for health information were found: (1) to obtain reassurance, (2) to seek a second opinion, (3) to improve understanding, and (4) the ease of online search compared with using traditional sources [7]. The scale of health and medical-related online search and the importance of the outcomes in terms of informing and influencing patient behavior mean that it is important to gain a detailed understanding of the online search process [7,8]. Prima facie, techniques and approaches used in consumer markets [9] have significant potential in contributing to our understanding of health and medical online search behavior. That is, research concepts and frameworks that have been used in other sectors to measure and evaluate online search behavior can be adapted and used in a health care context.

Although the Internet has greatly reduced consumer search costs, the scale and complexity of the available information requires sophisticated search methods by patients [8,10]. In this context, Google in particular has been described as the “gatekeeper to Web information,” due to its dominance as a search engine, directing searchers to a selected set of websites [11-13]. However, many users still feel that they need help in searching for information [13] and in evaluating the trustworthiness and veracity of online health and medical information [14,15]. These search issues are exacerbated in an eHealth search context because it often concerns an unfamiliar and unknown problem, where the absence of well-defined keywords initiates a series of cognitive learning and reformulating processes [16].

The use of the Internet by patients has the potential to significantly increase patient informedness and is clearly an important part of a health care philosophy of patient-centered medicine, where the patient is placed at the center of decisions and treatments. Such informed patients actively engage in their health management, understand their conditions and diseases, and discuss the medical decision making with their health care professionals. Medical information that was previously exclusive to members of the medical profession is now much more widely available [17,18], a trend that has encountered some professional resistance. There are concerns over the accuracy and validity of the information, the patients’ ability to understand the information, and also to be able to discern the quality of competing information sources. Given the scale, complexity, and extent of eHealth search, it is therefore important to gain a better understanding of the overall picture of eHealth search by examining a large sample of eHealth websites in terms of their purpose to develop a taxonomic structure and also by evaluating the detailed search patterns by patients or consumers on individual websites.

The following 4 main gaps have been identified in the literature:

First, very little information about the landscape of eHealth websites exists, that is, a high-level view of the most important websites measured by scale in terms of their categorization and usage. This is important because it would inform medical professionals and patients about the structure of website information defined in a taxonomy and would provide valuable insights into the scale and allocation of search effort by patients.

Second, there is very little research into the detailed measurement of eHealth search behavior on a large scale, raising the question of how eHealth search behavior can be analyzed and measured in a systematic and objective manner, using techniques and methods from other online markets [9,10].

Third, how do consumers actually navigate the eHealth landscape? That is, their search patterns have not been established.

Fourth, the differences between online behavior in private (United States) and public (United Kingdom) health care markets have not been investigated. This is an important issue for health policy because it starts to inform the research agenda of how private and public health care systems with different characteristics, for example, restricted versus universal access to health professionals and regulatory differences for advertising, affect online search behavior. These gaps are described in more detail below.

### The eHealth Landscape

Previous research has investigated the influence of search engines on the structure of the eHealth landscape [12,13] and surveys reveal the approximate scale of usage in terms of the number of eHealth users and frequency of use [15], but there is very little research on mapping out the structure of the eHealth landscape in terms of different types of eHealth website and their scale. Although there have been some efforts to develop classifications [4,19], there was no attempt to evaluate the models and populate them with a large-scale realistic dataset and there is no agreed classification or taxonomy of health-related websites.

### Measurement Framework

Previous research into eHealth behavior can be grouped into two categories: intensive research methods such as focus groups, experiments, and observational studies [3,10,20] and more extensive methods such as surveys [1,5]. Intensive research methods generate a high level of detail and allow the researcher to explore the motivation and logic of particular actions such as search strategies. They are based on relatively small samples of users, typically around n=20, which raises the problem of generalizability. This is exacerbated if the sample is of a specific nature; such as patients having a particular condition or being members of a professional group [3,6]. In addition, such samples are often from a single country [19,21]. Surveys provide potentially much larger samples and better generalizability, but there are also problems, notably, the difficulty of balancing the length and complexity of the survey with the practicalities of users completing it in a comprehensive and accurate manner [22]. There is also a general issue of the accuracy of self-reported behavior in surveys. To overcome these issues in an eHealth context where the purpose of the research is to try and capture highly accurate eHealth search behavior from a very large sample, online panel data are used. Göritz et al [23] explained the use of online panel data in a social science context in an authoritative, general discussion. The methodology, ideas, and concepts used in this paper are adapted from marketing where these techniques have been more widely used [9,24,25]. This enables the accurate measurement of variables such as the number of unique visitors who use a set of websites, the time spent per website, and the breadth of the search process measured by the number of different websites accessed [26,27].

### Patient Online Search Patterns

In general, there is very little systematic research and understanding of how consumers actually search for eHealth information in terms of their specific search patterns based on a very large sample of users. Previous research into eHealth search behavior tended to focus on very small groups of users, specific websites, or on the characteristics of the searchers themselves [4,15,28,29]. The bigger picture is therefore largely missing, except from surveys which capture the volume of activity based on self-reported online activities but not the related search behavior across multiple websites, for example, the number of websites visited within a related set of websites or detailed information such as the amount of time spent per website.

### Differences Between the Private and Public Health Care Markets

One might reasonably expect privately funded eHealth users to be more engaged in their eHealth activities because of the financial motivation. An additional factor, direct to consumer advertising of drugs is also expected to stimulate patient interest, which would be reflected in more intensive online search patterns, as compared with those of users in a public market. The approach taken in this study is to analyze the US and UK markets as exemplars of private and public health markets. These countries are similar in that both have (1) high levels of Internet penetration, (2) sophisticated use of online technology across all market sectors, and (3) advanced health care systems. The main points of difference are in the nature of the funding, where the United States is predominantly a private system compared with the United Kingdom, which is mainly public [30], and the United States allows direct to consumer advertising of drugs, whereas it is prohibited in the United Kingdom and also in Europe. The two markets are significant from an economic perspective because the United States is the largest pharmaceutical market, and the United Kingdom is part of the second largest pharmaceutical market worldwide, the European Union. Previous studies of online search in health markets found that language strongly affects the behavior [5], warranting the United Kingdom as the best choice within the European Union. In addition, the vast majority of health content on the Internet, regardless of country, is in English [31].

### Research Questions

1. What is the structure of the eHealth landscape described as a taxonomy of eHealth websites?
2. How can eHealth search patterns be analyzed using an objective measurement framework?
3. What are the eHealth search similarities and differences between public and private health care markets?

## Methods

### Taxonomy

The taxonomy was developed using a combination of conceptual development based on categories from the literature and was then adapted and extended through empirical experimentation with a sample of the largest 100 eHealth websites taken from the comScore commercial database [25,32]. The size of the websites was measured by the number of unique visitors per month, which ranged from 15,000 to 10 million in the United Kingdom and 45,000 to 70 million in the United States.

### Online Panel Data and Measurement Framework

The accurate measurement of online behavior requires objective data with international scope, very large scale, detailed granularity, and tracking ability across multiple websites. Online panel clickstream data were used because it fulfills all these data criteria [24-27]. “Clickstream” data recorded electronic data of Internet usage which was collected automatically from a panel of online users [33-35]. Online panel data are more reliable than interviews and questionnaires because it does not rely on self-reporting of Internet usage, and the automatic data collection can be implemented on a very large scale, which gives statistical reliability. It has been widely used in market research [9,24,33-36] and can also be used to make direct comparisons between international markets. In this research, the largest commercial international panel from comScore has been used, which tracks 1.5-trillion digital interactions monthly from more than 2-million registered users in 172 countries who access 3-million websites [25]. The data are gathered through a program installed on the computers of registered users which records the URLs of all pages viewed, how long a window has been active, and the actual pages viewed [26,27]. All data collected are encrypted to ensure complete privacy protection of users, and no personally identifiable information is released. A list of the websites used in the study is provided (Multimedia Appendix 1).

comScore tracks all types of websites, and the database of websites that are tracked is in effect defined by the users and is entirely dependent on the users’ online behavior. The company then categorizes the database, which means that all health- and medical-related websites of significant size are tracked [27]. The more important limitation of the research sample is therefore in the selection of the websites by the researchers. The research sample was an iterative process in which the starting point was to use comScore’s own categorization of health and medical websites and then to refine it using the expertise of the researchers and their colleagues. A small number of additional websites were added, including two important US Government websites. There are two possible sampling errors in the dataset used in this study: the inclusion of irrelevant websites and the exclusion of important websites.

The first error is mitigated by checking and categorizing each website according to the proposed taxonomy. The second type of error cannot be removed altogether although it is mitigated by the large sample size and the sampling process. The large size of the sample means that it is likely that the data are representative of usage and that the results are therefore robust. The statistical significance of differences is also estimated and because of the very large user samples of 1 million in the US and approximately 80,000 in the UK, the measurement errors are very small. Refer to previous studies [1,23,24,36] for examples of how comScore has been used by other researchers and Government bodies. A detailed methodological discussion of the general use of online panels in research that covers panel recruitment, composition, and validity of the data is given in the following core texts [37,38].

A multilevel framework was used to capture different aspects of search. Each measurement is defined in the following conceptual terms.

#### Search Penetration

The simplest and arguably the most important measurement is search penetration: this is defined as the overall level of search within a defined market compared with the country’s Internet population, that is the percentage of the Internet population that accesses eHealth content. Search penetration is reported for each category of eHealth website. It is important because it describes the overall level of market adoption of health-related search and represents the level of interest in health topics.

#### Allocation of Search Effort Across Categories

The composition of the search is defined as the distribution of users across categories of eHealth websites. It is important because it describes the allocation of search effort across categories and the relative size of each category.

#### Single Preferred Website and Searcher Split

This measure captures the distinction between those users who look exclusively at one, preferred website and those who search across two or more websites within a category. Those looking at a single website are likely to be accessing information from their preferred website, whereas those looking at more than one website are actively conducting search based on a consideration set of eHealth websites.

#### Consideration Set

A consideration set is the number of websites a user visits to conduct his search. Those looking at two or more websites are most likely to be actively searching within a set of related websites, and the consideration set concept is used to measure the range of websites that are accessed [9,24].

#### Time Per Visitor Per Website

This measure indicates the average length of time spent per website. It is a measure of the intensity of the search process and is a good proxy for search effort or engagement per individual website [34].

#### Private and Public Index

For each of the variables, search penetration, consideration set, and time per visitor per website, a private and public index is defined as the ratio of the US:UK result. This gives a comparative measure of the development or use of the Internet in a private market compared with a public one.

## Results

### Taxonomy of eHealth Websites

The proposed taxonomy of websites is shown in Table 1.

The proposed taxonomy is based on the synthesis of individual categories identified in the literature, in particular, health [39], medical [15], lifestyle [40], e-pharmacy [41], social media [6], and professional [42]. This new eHealth taxonomy is broader in scope and more comprehensive than similar frameworks [4,19]. Although the classification scheme proposed by Di Giacomo et al [19] has some similarities to our model, it did not identify the health, lifestyle, and social media categories. The proposed taxonomy has therefore been derived in a mainly deductive manner and then applied empirically to a large sample of websites. The categorization of the empirical data indicates that the taxonomy appears to be comprehensive and useful, that is, the researchers could place all the researched websites naturally into an individual category. Our approach is therefore similar to the methods for developing taxonomies that combine both deductive and empirical approaches [32].

**Table 1:** Health website categories, their general description, and subcategories.

Category	General description	Subcategories	Number of websites
Health	Broad information about health encompassing a wide variety of information centered on everyday health issues.	General health information	20
		Health news	
		Women’s health	
		Children’s health	
		Men’s health	
		Other health information	
Medical	Websites providing specific information about diseases, conditions, treatments and symptoms.	General medical information	26
		Drug information	
		Services, physician and hospital information	
		Specific disease information	
		Mental health and psychology	
Lifestyle	Information websites centered on well-being and healthy living.	General healthy lifestyle information	20
		Natural health and alternative medicine	
E-pharmacy	Websites providing information and products for medication.	Online pharmacies	20
Social media	Platforms dedicated to communication among patients, peers, and professionals to interact and discuss health and medical issues.	Social media platforms	4
		Health forums	
Professional	Websites providing information specifically targeted to health care professionals.	Information for health professionals	10
		Pharmaceutical companies	

#### Search Penetration

Online search penetration is an important measure because it is a proxy for the level of interest and awareness in a subject. Online penetration is an adaptation of the standard concept of market penetration from marketing and is an indication of the maturity of the market measured by the product lifecycle [43]. The number of unique visitors and search penetration in each category is shown in Table 2. To take out the effect of market size, the results are calculated as a search penetration percentage of the total online population in each country. The total Internet population is 251 million (US) and 48 million (UK) [44]. The search penetration index is the private (US):public (UK) ratio of the search penetration of eHealth websites in each eHealth category.

Health is the largest category in both markets and the search penetration index is a factor of 2, which is an enormous difference. Lifestyle shows a similar level of difference between the two markets. These two categories are general in nature and demonstrate a much higher level of interest in the private than the public health care market for general health topics. Medical search penetration is identical, and this may reflect the fact that very specific medical search originates from an interaction with a health care professional rather than from general interest in a topic. The level of search into e-pharmacy websites is relatively low in both the countries compared with other categories. The US e-pharmacy penetration is significantly higher than the UK, which is perhaps a reflection of direct to consumer advertising of prescription drugs. The exception to the general pattern is professional websites, where a much higher proportion of UK users than US users access these websites.

#### Allocation of Search Effort Across Categories

The search distribution shows the relative size of each category relative to the other categories within each health care market and the results are shown in Table 3.

The distribution of overall search effort is quite similar and shows that health, medical and lifestyle account for around 90% of Internet activity in both markets. The exception is professional, which accounts for a higher proportion of overall UK activity compared to the US.

#### Single Preferred Website and Searcher Ratio

In general across all categories, most online users only access one website within each category (Table 4). The highest level of search is in health even in which only 22% of all users look at two or more websites. These results are consistent with previously reported levels of search in online consumer markets [34,35,45]. The category with the lowest level of searchers is the e-pharmacy category, which suggests that almost all patients use a single, preferred e-pharmacy. Considering the nature and impact of eHealth content, it is also likely that consumers are risk averse and therefore contain their online activity to a single, trusted website.

#### Consideration Set

The consideration set is relatively narrow in all cases, particularly when considering the wide choice of websites within each category (Table 5) and the two markets are almost identical. Narrow search patterns are the norm in other consumer markets [9,24,34,45]. In the health market, it is reasonable to assume that the complexity of the information naturally limits search patterns because of the effort taken to read and comprehend the information.

**Table 2:** Search penetration: unique visitors (millions) and search penetration index.

Category	Unique visitors (millions) and search penetration (%)		Search penetration index
	United Kingdom (public HCM^a^)	United States (private HCM)	
Health	19.8 (41%)	207.7 (83%)	2.0
Medical	16.8 (35%)	90.8 (36%)	1.0
Lifestyle	5.4 (11%)	60.8 (24%)	2.1
E-pharmacy	0.7 (1%)	7.1 (3%)	2.0
Social media	0.9 (2%)	14.6 (6%)	3.1
Professional	4.9 (10%)	9.6 (4%)	0.4

^a^HCM: health care market.

**Table 3:** Distribution of search effort.

Category	Public HCM^a^	Private HCM
Health	41%	53%
Medical	35%	23%
Lifestyle	11%	16%
E-pharmacy	1%	2%
Social media	2%	4%
Professional	10%	2%

^a^HCM: health care market.

**Table 4:** Single preferred website and search.

Category	Single website		Search	
	Public HCM^a^	Private HCM	Public HCM	Private HCM
Health	78%	75%	22%	25%
Medical	87%	84%	13%	16%
Lifestyle	88%	85%	12%	15%
E-pharmacy	92%	98%	8%	2%
Social media	91%	93%	9%	7%
Professional	95%	93%	5%	7%

^a^HCM: health care market.

**Table 5:** Results of consideration set.

Category	Public HCM^a^	Private HCM	Consideration set index
Health	2.4	2.3	1.0
Medical	2.2	2.3	1.0
Lifestyle	2.3	2.3	1.0
E-Pharmacy	2.1	2.0	1.0
Social media	2.0	2.1	1.1
Professional	2.1	2.2	1.0

^a^HCM: health care market.

**Figure 1:**
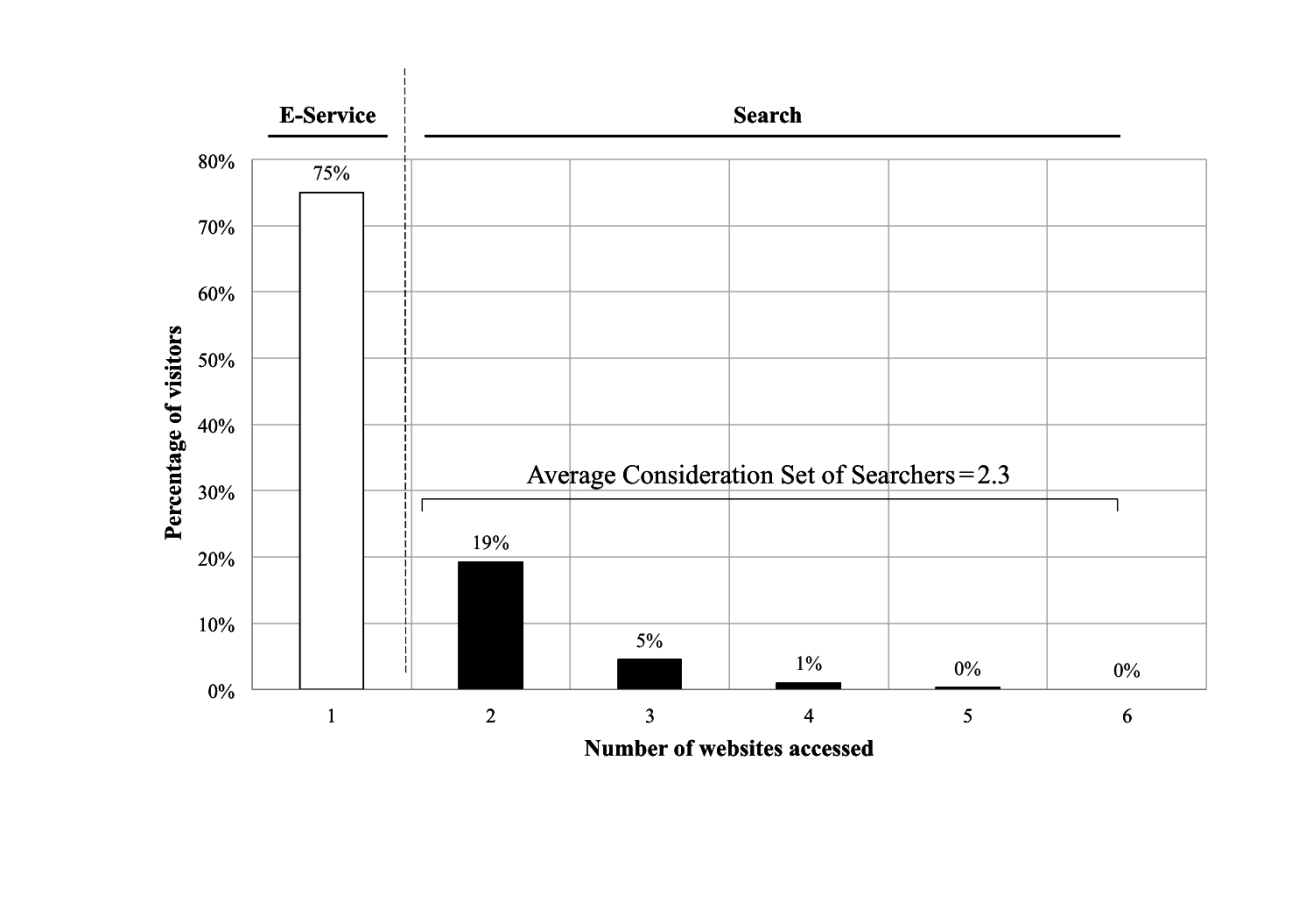
The distribution of all visitors in the private (US) health category.

**Figure 2:**
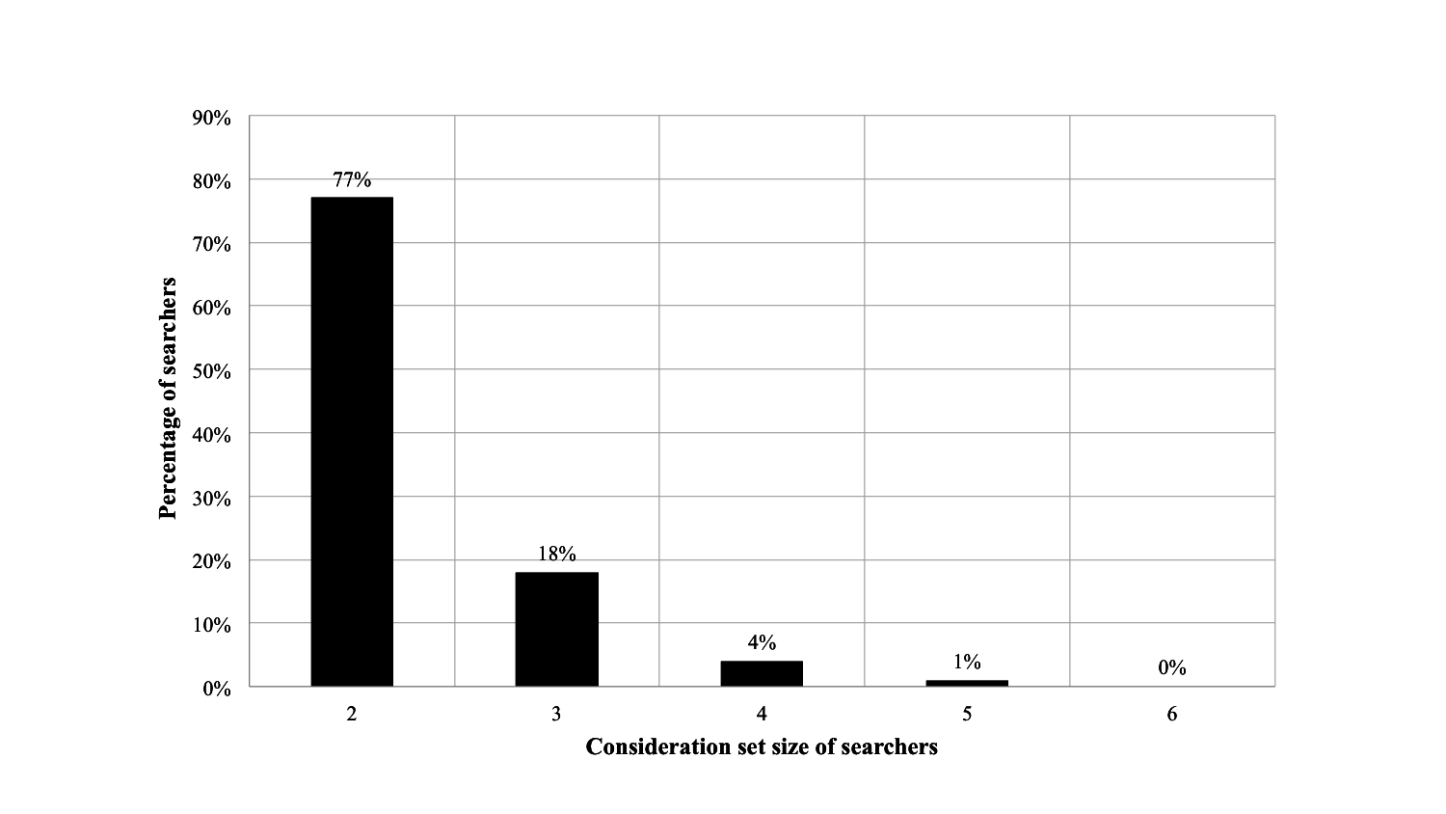
The distribution of searchers in the private (US) health category, average consideration set size=2.3.

Note that this is an average consideration set. To understand its composition better, the distribution of all visitors is given in Figure 1 and for searchers only is given in Figure 2 for the private (United States) health category.

The distribution of all visitors (Figure 1) shows that the split between single website and searchers is 75:25%. The distribution of the searchers only (Figure 2) shows that 95% of searchers look at just two or three websites, with only 5% conducting what could be termed an extensive search pattern of four or more websites.

#### Time Per Visitor Per Website

The average time spent per website per unique visitor is a measure of the depth of the search process. It is therefore a measure of engagement or attention between the user and the website content. The results are shown in Table 6.

Patients in the private health care market are more engaged across all categories of eHealth content, and this is possibly due to the financial risk and costs of private health care. A more engaged search process could therefore be interpreted as a risk reduction strategy. Another explanation is that users in the United Kingdom may have better and easier access to health care professionals than in the United States, which gives all users, regardless of income, an alternative to online search.

#### Sample Size and Statistical Significance

A sample size table was calculated based on the number of visitors in each category, the size of the online panel, and the overall digital population in each country. The estimate of unique visitors is equal to the number of panel members who visit a set of websites (ie, the sample size for each category in the taxonomy) as a proportion of the online panel size multiplied by the digital population. The results for each category are shown in Table 7.

The sample sizes range from 1100 to 828,000. In the United Kingdom, the sample sizes for health, medical, lifestyle, and professional are of the order of 10,000, and 1000 for e-pharmacy and social media. In the United States, the smallest sample is 28,200. With samples of this magnitude, differences between categories are almost certainly real. Search penetration in Table 2 and average time per visitor in Table 6 represent arguably the two most important measures of search effort, that is, the level of usage measured by penetration of the population, which is equivalent to the concept of adoption in marketing, and then average time spent per website per user, which is a measure of search effort or engagement that is independent of market size. The differences between the United Kingdom and the United States for the variables search penetration and average time per website across each of the categories are all significantly different, *P*<.001.

The very low *P* values can be attributed to the scale of the differences between the samples and the very large sample sizes. It is therefore essential and useful to also consider the effect sizes to give a complete picture of the implications of the results and relate them to the differences in the behavior of digital populations that are independent of the sample size [46]. The effect sizes were measured using odds ratio (OR) for search penetration and Cohen *d* for time per website [46]. The results are shown in Table 8.

**Table 6:** Average time per website per unique visitor in minutes.

Category	Public HCM^a^	Private HCM	Time index
Health	3.13	4.36	1.4
Medical	3.14	3.42	1.1
Lifestyle	3.91	4.82	1.2
E-pharmacy	3.20	5.56	1.7
Social media	2.84	3.16	1.1
Professional	3.08	7.31	2.4

^a^HCM: health care market.

**Table 7:** The sample size estimate of each category for the United Kingdom and the United States (represented in thousands).

Category	Public HCM^a^	Private HCM
Health	32.9	827.6
Medical	27.9	361.8
Lifestyle	9.0	242.1
E-pharmacy	1.1	28.2
Social media	1.5	58.3
Professional	8.1	38.4

^a^HCM: health care market.

**Table 8:** Effect sizes for search penetration and time per website per visitor.

Variable	Search penetration OR (95% CI)	Time per website Cohen *d*
Health	2.01 (1.99-2.04)	0.37
Medical	1.04 (1.02-1.05)	0.02
Lifestyle	2.15 (2.10-2.20)	0.24
E-pharmacy	2.05 (1.93-2.18)	0.82
Social media	3.11 (2.95-3.27)	0.15
Professional	0.38 (0.37-0.39)	0.98

The OR was used to measure the effect size for search penetration because it is a binary outcome, that is, adoption or non-adoption of Internet search within a particular category of eHealth. The effect size interpretation used is small (1.5), medium (2.0), and large (3.0) [46]. For search penetration, the results show a medium effect size for health, lifestyle, and e-pharmacy, a large effect size for social media and a very weak effect size for medical. The effect is in the opposite direction for professional, and in terms of its magnitude is medium. For Cohen *d*, the thresholds of small (0.2), medium (0.5), and large (0.8) are adopted [46]. For time per website, large effect sizes exist in e-pharmacy and professional and small effect sizes in health and lifestyle. The time effect size is very weak for medical and social media.

Health and lifestyle categories are both large categories within the eHealth taxonomy (see Table 3). The effect sizes for search and time in these categories therefore represent important search differences between the US and UK health care markets. The very small effect sizes in the medical category confirm the strong similarity between the UK and US health care markets for medical search.

In e-pharmacy, the results may indicate a higher level of maturity for online ordering of prescriptions in the United States compared with the United Kingdom. The social media category represents just 2% and 4% of overall search effort in the United Kingdom and United States, respectively (see Table 3), although its use could grow as eHealth users become more confident and Web 2.0 technology improves. The effect size for search penetration in the professional category is opposite to the other categories and this requires further research.

## Discussion

### Principal Findings

A context is needed to measure online behavior in any market. In the case of eHealth, the most suitable framework was to develop a taxonomy to allow grouping of similar websites into health, medical, lifestyle, e-pharmacy, social media, and professional. A measurement framework was also developed to measure search behavior in a holistic manner, which took into account five key aspects of the search process: (1) search penetration, (2) allocation of search effort across categories, (3) distinguish between single website use and search across two or more websites, (4) Consideration set within each category, and (5) time per visitor per website. The application of the measurement framework in the context of the taxonomy enabled us to compare a private (United States) and a public (United Kingdom) health care market in a much more sophisticated and nuanced manner than simple measures such as the number of online users.

The search penetration indices for health and lifestyle for the private market are twice as high as the public one. On the basis of Internet adoption rates, the private market is therefore much more developed than the public one. For both health and lifestyle, consumers of the private market also spent more time per website. The evaluation of market penetration and time together indicates that users in the private market are generally more interested and also more engaged in eHealth content. Note that these differences cannot be attributed to general differences in Internet sophistication, which are very similar by other measures as reported by authoritative sources [44,47,48]. The exception to search penetration is the professional category where UK Internet use is more extensive in terms of search penetration. This implies that UK users may be more sophisticated in searching this highly specialized content, where the specialized and detailed nature of the information also appears to limit search because 95% of UK and 93% of US users only access one website in this category. These conjectures require further research, which could focus on this category only and perhaps explore the use of a larger number of websites coupled with qualitative data about online search behavior.

The search behavior in the medical category is almost identical, which begs the question why online users in two very different health environments should behave so similarly. One explanation is that medical search is very specific, that is, it relates to a condition or treatment. In this context, it is much more likely that the search is by patients, or their carer, responding and reacting to a specific need for further information and advice on a medical topic. The stimulus for the search process is therefore most likely to be as a result of a medical consultation with a professional, or a patient informing themselves of the likely diagnosis or treatment of an ailment. In this particular medical context, the main factor initiating the search process is likely to be a medical event, which is equally likely in both countries.

The allocation of search across categories has the same rank order, where health, medical, and lifestyle websites attract approximately 90% of online activity in both markets. Social media websites are very small in comparison with the professional websites in health, medical, and lifestyle and may indicate that eHealth users rely more on professional content rather than peer advice.

The analysis of single website and search showed that the majority of online users, between 75% and 98%, only look at one website within each category. This implies that they have a preferred, single source of information rather than searching across multiple websites for information. Those who look at two or more websites have relatively narrow search patterns. These results are consistent with other markets [9,35].

The consideration sets are almost identical for both markets. The most interesting result is that the search process is very narrow and it is shown in Figure 1 that even for the largest consideration set of 2.3, 95% of users only look at 2 or 3 websites, that is, only 5% of users conduct an extensive search process. This has important implications because it suggests that most users do not assess multiple websites and rely on a handful of very large, influential websites within each category. This assertion is supported by an inspection of the eHealth website sizes shown in Multimedia Appendix 1, measured by the number of unique visitors over a fixed period of time. The average time per website for each user is just a few minutes, with private users spending more time on health and lifestyle categories. This indicates a shallow search process and raises important questions about the level of patient informedness.

**Figure 3:**
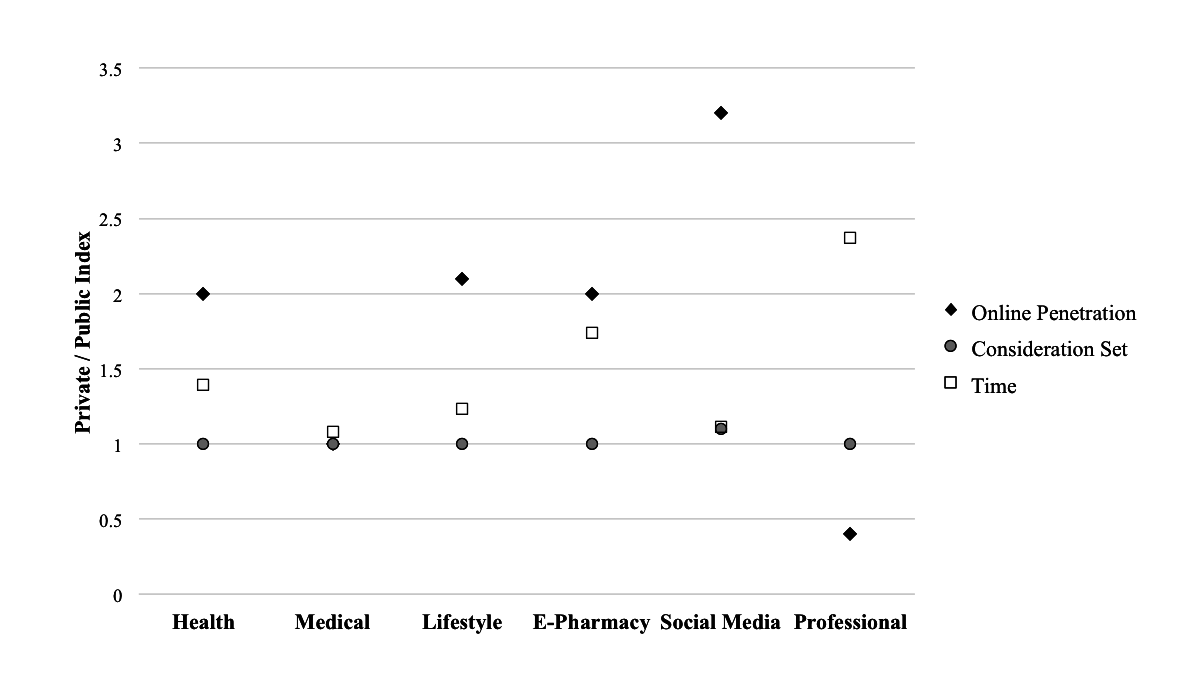
Online indices for online penetration, consideration set, and time per website, calculated by dividing private score by public score in each category.

A synthesis of the results is shown in Figure 3. Each score is a result of the private and public index ratio for each of the measures of online penetration, consideration set size, and the average time spent per website. Online penetration is a direct measure of overall adoption of the Internet; consideration set is a measure of the breadth of search; and the time spent per website is a measure of user interest, or engagement, in the website content. These results show that the private health care market has significantly higher adoption rates for eHealth websites in the health, lifestyle, e-pharmacy, and social media catagories measured by online penetration and that they spend significantly more time on health, lifestyle, e-pharmacy, and professional websites. The consideration sets are almost identical for all categories. The medical category is the same for all measures, which suggests that other factors such as interaction with professional medical staff and medical conditions determine the nature of the search process for specific medical information that probably relates to a specific condition or ailment.

In general, the private US market is more developed in terms of eHealth search than the public UK market. Given that both markets are quite similar in general e-commerce terms [1,44,47], the most plausible explanations are (1) that the private health care system incentivizes personal search into eHealth to encourage general health and well-being; and (2) free access to health care professionals is more widely available in the United Kingdom compared with the United States, including visits to physicians and health care support through telephone.

### Limitations and Future Research

The United Kingdom and United States were chosen as representative countries because they are similar in terms of their demographic structure, economy, and Internet access and usage [1,44,47]. Crucially, the two countries share the English language, eliminating effects of language on search behavior. It is therefore plausible that the observed differences can be explained by the differences in funding of the health care systems, where US patients are personally responsible for organizing and paying for their own health care. Other factors such as the availability and ease of access to physicians were also considered, and both of these factors require further research to understand the complex interactions between the online channel and face-to-face meetings between health care professionals and patients.

The analysis in this study was concerned with the whole of the digital population in each country. Previous research has identified the importance of user characteristics and more broadly the concept of eHealth literacy in influencing search behavior [39,49], and there is potential for developing the analysis in this study by analyzing and exploring the behavior of specific demographic groups and also to make further international comparisons.

Clickstream data provides very detailed information about search patterns, for example, the consideration set concept and the exact amount of time spent per website is captured, which cannot be measured as accurately or on such a large scale using a traditional survey of eHealth trends [5]. These types of results are important for overall eHealth policy making [50] and also have implications for eHealth design [51]. However, a limitation of clickstream data is that the results only measure the actual behavior of users and do not inform us of their motivations, that is, it is not known why they pursue a particular course of action. One way of compensating for this weakness is to conduct a parallel online survey of the panel members that is more qualitative in nature and this is an interesting area for future research.

### Comparisons With Prior Work

The proposed taxonomy framework is similar to that of previous study [19], although we think it is important to have more granular categories to uncover important differences in search behavior, which has been supported by the empirical results that identify significant variations and also important similarities in the way that the US and UK health care populations access eHealth information. The diffusion of eHealth adoption is an important topic because it tells us about how the general population is accessing health and medical information [50,52-54] Research using large surveys of eHealth use [5,53] provides very good general information about the use of particular websites and can be used to estimate the online adoption rates and factors that influence eHealth use within a population. However surveys cannot accurately measure detailed outcomes such as the consideration set concept or time spent per brand, and the approach used here is novel in this respect. The approach taken in this study has been to assess the demand side of eHealth usage combined with the categorization of the supply side of eHealth websites, and this is important because it leads to a better understanding of actual usage that can inform policy [50]. Previous research has shown that a country’s health care system and a person’s insurance status have little effect on patients’ online search behavior for health information [1,55], which is not supported by our results.

### Conclusions

The taxonomy of eHealth websites was developed using a combination of deductive and inductive methods and is a useful way of describing and categorizing eHealth websites. The online measurement framework is an important step toward a standard approach to measuring eHealth search behavior, similar to other standard health care measurement systems that are used in health surveys [22]. Online panel data provide a reliable source of data that can be used to conduct standard measurement between different segments of the population, between countries and also to enable longitudinal studies to assess important changes and trends over time.

There are significant statistical differences measured by *P* values and also effect sizes [46] for search penetration and time per website in the health and lifestyle categories. One possible explanation is that patients in the United States have a personal financial incentive because of the private health care system whereas in the United Kingdom, the service is free. Another explanation is that the availability of health care information through health care professionals is more widely available to all patients in the United Kingdom, regardless of income. This explanation is supported by data from the British Medical Association and the US Department of Health and Human Services, which shows that the average number of visits to a physician in the United Kingdom is 6 times per year, twice that of the United States [56,57]. In addition, there is an extensive telephone support system supported by the National Health Service (NHS) in the United Kingdom, which does not have a similar counterpart in the United States. The more general point here is that the availability of health care information through other channels, for example, health care professionals, television and printed media, may reduce the adoption of the Internet channel [58].

The allocation of search effort across the categories is very similar for the private and public markets and show that almost 90% of Internet activity is accounted for by the health, medical, and lifestyle categories. Social media focused eHealth websites are very small and users prefer authoritative content from professionally published websites rather than accessing information from their peer group.

In all categories of eHealth, between 75% and 98% of users access one website only, which suggests that users find a trusted source and remain loyal to it. Consideration sets are relatively small and fall within the range 2.1-2.3. These narrow search patterns may reflect the complexity of the information and perhaps risk-averse search behavior, that is, most searchers evaluate just two or three websites. The time spent per website is also relatively small, although higher in the US. This result suggests that the overall level of informedness will be low, given the low level of search effort measured by time per website and the fact that within each eHealth category most users access just one website and most searchers only visit two or three websites.

## Appendix

Multimedia Appendix 1 eHeath website classification and unique visitors (represented in thousands; source: ComScore).

## Abbreviations

EU: European Union
HCM: health care market
OR: odds ratio
UK: United Kingdom
US: United States
