# Supplementary material for: eHealth Search Patterns: A Comparison of Private and Public Health Care Markets Using Online Panel Data
Source: J Med Internet Res. 2017 Apr 13;19(4):e117. doi: 10.2196/jmir.6739 (PMC5408137; doi:10.2196/jmir.6739)
Supplement: Multimedia Appendix 1 [file jmir_v19i4e117_app1.pdf]

		Unique Visitors (000s)	
Website name	Classification	UK	USA
About.com Health	Health	1,965	18,404
BBC News Health	Health	4,441	2,430
CNN Health	Health	84	25,628
Discovery-Life	Health	308	2,408
EmpowHER	Health	45	1,378
Everyday Health	Health	2,833	48,075
HEALTH.COM	Health	624	6,098
HEALTHCARE.GOV	Health	-	1,334
Healthline	Health	1,600	15,986
Hearst UK Wellbeing Network	Health	2,426	3,044
KIDSHEALTH.ORG	Health	270	2,499
LIFESCRIPT.COM	Health	85	14,297
MED-HEALTH.NET	Health	76	302
MEDICALDAILY.COM	Health	88	910
MEDINDIA.NET	Health	74	575
Nytimes Health & Science	Health	328	6,062
Telegraph Health	Health	833	908
Today Health	Health	97	6,294
WebMD Health	Health	6,416	70,035
Yahoo Health	Health	38	12,328
ALLABOUTVISION.COM	Medical	97	639
AMERICANHEART.ORG	Medical	74	1,574
APA.ORG	Medical	128	794
CALMCLINIC.COM	Medical	57	162
CANCER.ORG	Medical	106	1,839
DISABLED-WORLD.COM	Medical	61	219
DRUGS.COM	Medical	1,462	22,179
HEALINGWELL.COM	Medical	103	545
Healthgrades	Medical	1,181	25,908
HEALTHHYPE.COM	Medical	115	81
HEALTHYPLACE.COM	Medical	25	193
HEALTHYREPLY.COM	Medical	104	119
LABTESTSONLINE.ORG	Medical	51	593
MD-HEALTH.COM	Medical	108	686
MENTALHELP.NET	Medical	15	89
MERCKMANUALS.COM	Medical	114	954
NEWHEALTHGUIDE.ORG	Medical	175	1,054
NHS Sites	Medical	10,108	993
NIH US Government	Medical	1,660	9,649
PSYCHCENTRAL.COM	Medical	478	4,396

Remedy Health Media	Medical	304	6,032
SOCIALANXIETYSUPPORT.COM	Medical	67	449
SPINE-HEALTH.COM	Medical	142	1,051
SYMPTOMFIND.COM	Medical	116	2,263
TREATO.COM	Medical	117	453
Vitals Patient Exch.	Medical	914	13,370
ALLFITNESSWEB.NET	Lifestyle	68	402
BEYONDDIET.COM	Lifestyle	67	1,476
FITNESSMAGAZINE.COM	Lifestyle	239	3,015
GREATIST.COM	Lifestyle	274	3,360
HEALTHYFOODHOUSE.COM	Lifestyle	49	699
IHERB.COM	Lifestyle	59	430
India.com Health	Lifestyle	101	626
iVillage Health	Lifestyle	195	2,391
IVY-ROSE.CO.UK	Lifestyle	45	45
LIVESTRONG	Lifestyle	2,602	26,857
LoveToKnow Health & Beauty	Lifestyle	102	565
MERCOLA.COM	Lifestyle	169	2,538
MSN Health & Fitness	Lifestyle	493	7,412
MUSCLEANDFITNESS.COM	Lifestyle	315	2,489
NATURALBREAKTHROUGHSRESEARCH.COM	Lifestyle	30	487
NATURALCURESNOTMEDICINE.COM	Lifestyle	45	339
Prevention Magazine	Lifestyle	327	4,179
Sparkpeople	Lifestyle	407	7,302
TOP10HOMEREMEDIES.COM	Lifestyle	145	832
WELLNESSMAMA.COM	Lifestyle	33	1,157
LLOYDSPHARMACY.COM	e-pharmacy	281	-
VIOVET.CO.UK	e-pharmacy	125	-
PHARMACY2U.CO.UK	e-pharmacy	114	-
HYPERDRUG.CO.UK	e-pharmacy	43	-
PETMEDS.CO.UK	e-pharmacy	41	-
BODYKIND.COM	e-pharmacy	30	-
UK-ONLINE-STORE.COM	e-pharmacy	27	-
121DOC.CO.UK	e-pharmacy	19	-
HEALTHEXPRESS.CO.UK	e-pharmacy	17	-
GOODRX.COM	e-pharmacy	-	1,945
Express Scripts, Inc.	e-pharmacy	-	1,708
HY-VEE.COM	e-pharmacy	-	662
CAREMARK.COM	e-pharmacy	-	610
NAMI.ORG	e-pharmacy	-	597
Walmart Pharmacy	e-pharmacy	-	398
MEDIXSELECT.TV	e-pharmacy	-	323
MEDCO.COM	e-pharmacy	-	296

AMPYRA.COM	e-pharmacy	-	233
DRX.COM	e-pharmacy	-	192
PEOPLESPHARMACY.COM	e-pharmacy	-	191
EHEALTHFORUM.COM	Social Media	136	1,504
HEALTHBOARDS.COM	Social Media	368	2,824
HEALTHTAP.COM	Social Media	121	2,387
Sharecare Network	Social Media	298	8,445
Bayer Group	Professional	55	233
B-I GmbH	Professional	19	244
JNJ.COM	Professional	35	280
ROCHE.COM	Professional	26	76
AAOS.ORG	Professional	62	486
BMJ.COM	Professional	218	344
DOCTORSLOUNGE.COM	Professional	105	289
EMIS	Professional	4,096	5,076
Internet Brands Health	Professional	251	2,430
NEJM.ORG	Professional	37	266
